# “Killing two birds with one stone” – a qualitative study on women’s perspectives on the dual prevention pill in Johannesburg, South Africa

**DOI:** 10.1186/s12905-024-03269-8

**Published:** 2024-08-22

**Authors:** Siyanda Tenza, Lydia Mampuru, Mpho Moji, Sihle Zulu, Lorna Begg, Irene V. Bruce, Krishnaveni Reddy, Barbara A. Friedland, Thesla Palanee-Phillips, Sanyukta Mathur

**Affiliations:** 1 Faculty of Health Sciences, Wits RHI, University of the Witwatersrand, Johannesburg, South Africa; 2 Center for Biomedical Research, Population Council, New York, NY USA; 3 Social and Behavioral Research, Population Council, Washington, DC USA; 4 Department of Epidemiology; School of Public Health, University of Washington, Seattle, USA

**Keywords:** Multi-purpose prevention technology (MPT), HIV prevention, South Africa, Pregnancy prevention, Qualitative research, End-users

## Abstract

**Background:**

HIV incidence remains high in South Africa, with ~ 60% of all new HIV infections among adolescent girls and women (Country factsheets HIV and AIDS Estimates, 2022). Oral pre-exposure prophylaxis (PrEP), approved for HIV prevention in South Africa since 2015, is hampered by low uptake and adherence, particularly among adolescent girls and young women (AGYW). Combining oral PrEP with oral contraceptives could increase PrEP uptake, persistence and address unmet needs for contraception. We investigated the acceptability of a dual prevention pill (DPP), combining oral PrEP and a combined oral contraceptive (COC) for HIV and pregnancy prevention among women in Johannesburg, South Africa.

**Methods:**

Between March-July 2021, we conducted 12 focus group discussions (FGDs) with adolescent girls and women (*n* = 74) aged 16–40 stratified by ages (16–17, 18–24, 25–40), half of whom were COC users. We explored adolescent girls and women’s opinions about the DPP concept, existing HIV and pregnancy prevention options, and input on perceived facilitators and barriers to DPP use. FGDs were conducted in English or isiZulu, using a standardized interview guide. FGDs were audio-recorded, transcribed to English and analyzed using ethnographic content analysis.

**Results:**

The majority viewed the DPP favorably as a multipurpose option preventing unplanned pregnancy and HIV. Most saw it as a convenient “two-in-one” solution, requiring one clinic visit for both PrEP and COCs. AGYW were viewed as the most likely to benefit from the DPP due to the likelihood of multiple partners and unplanned sex, possibly preventing school dropout from unplanned pregnancy or HIV acquisition. The DPP was perceived to be more reliable than condoms, especially when condom negotiation is limited. Benefits were also seen by participants in rape cases, protecting against pregnancy and HIV. DPP use barriers included side effect concerns, unsupportive partners and judgmental healthcare providers.

**Conclusions/significance:**

The DPP was perceived as acceptable for HIV and pregnancy prevention to AGYW in Johannesburg and its dual indications helpful in supporting improved PrEP uptake and persistence. DPP implementation programs need to consider solutions to potential barriers, like education on DPP benefits, coupled with reliable side effect support and healthcare provider sensitization as part of routine sexual health services to encourage uptake and adherence.

## Introduction

Women and girls in South Africa experience high HIV incidence rates. The Evidence for Contraceptive Options and HIV Outcomes (ECHO) trial of contraception and HIV-1 risk (2019) demonstrated quadrupling of HIV incidence rates in women aged 15–35 years old and a rate of 5.03 per 100 woman-years (95%CI 4.10–6.12) among women aged 18–20 between 2015 and 2017 [1]. Adolescent girls and young women (AGYW) are considered a priority population for HIV prevention nationally. An estimated 13% annual increase in new infections is projected, to lead to 3.5 million new infections by 2030 among adolescents inclusive of girls in the absence of improved delivery of effective HIV prevention and care [2]. Although HIV incidence is highest among AGYW, several studies have indicated that older women are also at high risk of HIV [3, 4] and need access to acceptable HIV prevention options.

In December 2015, South Africa became the first African country to approve the use of Truvada (tenofovir disoproxil fumarate [TDF]/emtricitabine [FTC]) as oral preexposure prophylaxis (PrEP) for HIV prevention [5]. PrEP has the potential to reduce individual HIV risk and impact population-level HIV incidence when used consistently. The rollout of oral PrEP by the South African National Department of Health (NDoH) began in June 2016 and prioritizes AGYW, men who have sex with men (MSM), sex workers (SW), and sero-discordant couples [6]. Progress has been made toward achieving the annual targets in the National Strategic Plan (NSP) for MSM and SW; however, more effort is needed to reach AGYW, as only 66% of the 2018–19 targets have been met [7].

Although oral PrEP is highly effective in reducing HIV transmission [8], most PrEP trials and demonstration projects among women have encountered challenges with adherence, uptake, and consistent use – particularly among AGYW [9–11]. Stigma is often cited as a barrier for nonuse of PrEP [12–14]; women fear being regarded as HIV-positive or promiscuous if they take the same ARV drugs used to treat HIV. AGYW have voiced concerns about the consequences that PrEP use will have on their sexual relationships, as PrEP use signals mistrust and infidelity, which can potentially result in relationship dissolution or violence [15, 16]. Therefore, strategies are needed to expand HIV prevention options for women (including AGYW) at high risk of HIV.

Growing evidence indicates that women may be more apt to use an HIV prevention method if it also prevents pregnancy, i.e., a multipurpose prevention technology (MPT) [17–21]. Currently, the only available method for preventing both HIV and unplanned pregnancy is condoms. Male condoms, however, are not under a woman’s control, and female condoms have had limited uptake due to cost, access and acceptability issues (including male partners’ objections) [22]; many women risk gender-based violence by merely suggesting condoms [23, 24]. Several novel MPTs are in development, including a dual prevention pill (DPP) for HIV and pregnancy prevention [25, 26]. MPTs can be a valuable prevention tool for women who may have an unmet contraception need along with those who currently rely on condoms for HIV and pregnancy prevention but seek a more reliable and user-controlled prevention method. In one study, it was estimated that the DPP could double the number of women using PrEP in 15 African countries [27]. Beyond increasing PrEP uptake, MPTs such as the DPP can also potentially provide an affordable means for pregnancy and HIV prevention and could be particularly cost-effective for women who are not currently using any contraceptive method [28].

Combining PrEP with an oral contraceptive is likely to be the fastest route to an approved MPT to protect against unplanned pregnancy and HIV because it combines two licensed, marketed drugs. The first DPP in development is based on a 28-day contraceptive regimen – a combined oral contraceptive (COC) pill (150 mcg of LNG, 30 mcg of ethinyl estradiol [EE]) co-formulated with Truvada® (300 mg of tenofovir disoproxil fumarate [TDF], 200 mg of emtricitabine (FTC)] for HIV PrEP [26, 29]. COCs are widely available and are used by 5% of current modern contraceptive users in South Africa [30]. We hypothesize that DPP could greatly increase PrEP uptake and adherence. To that end, we conducted a study to gauge women’s interest in using the DPP, to gather their input regarding informational materials needed for DPP introduction and to understand their recommendations for appropriate service delivery settings.

## Methods

This qualitative cross-sectional study was conducted as part of the formative phase of a multi-phase acceptability study for the DPP in Johannesburg, South Africa.

### Study site

The DPP formative study was conducted at the Wits RHI Research Centre Clinical Research Site (CRS) in Hillbrow, Johannesburg, South Africa, a large purpose-built research clinic situated within the inner-city of Johannesburg’s Hillbrow Health Precinct. In Johannesburg, the HIV prevalence among AGYW is estimated to be 14.4% [31]. This urban regeneration zone spans 4 blocks and is characterized by a clustering of government, residential and university-related health facilities, and services.

### Participant recruitment

Participants were recruited from clinical and community-based settings, building on prior experience and relationships with local healthcare centers and community-based organizations. The researchers sensitized facility-level healthcare providers about the study and sought permission from the Johannesburg Health District Committee to recruit participants at select family planning (FP), primary health care, and HIV counseling and testing centers near the study site and surrounding areas. Both active and passive recruitment methods were employed. Active recruitment involved speaking directly with potential participants in waiting rooms and inviting those who were interested in the study clinic to learn more about the study. Passive recruitment involved leaving information about the study (fliers, posters, etc.) in clinic waiting rooms for women accessing reproductive health, HIV prevention, counseling and testing and contraception services to see and coming to the study clinic for more information if they were interested. For community-based recruitment, the local research team worked with the Wits RHI Prevention and Youth community advisory boards (CAB) to identify venues (including youth-based organizations) from which to recruit potential eligible participants.

### Data collection procedures

A total of 12 focus group discussions (FGDs) were conducted with women and AGYW. Six of these FGDs included women who currently use COCs, and six were among women who do not. The FGDs were further stratified by age groups (16–17-year-olds, 18–24-year-olds and 25–40-year-olds). Participant eligibility, aside from age was by self-report. Eligibility criteria included willingness to provide informed consent, current use of COCs for participants targeted for a COC user FGD, non-COC FGD participants had to be either using condoms, an injectable contraception or no contraception at the time. Additionally, participants had to be willing to be audio recorded as part of the FGD.

FGDs were conducted by trained social scientists at the Wits RHI CRS in a room dedicated to FGDs. One researcher facilitated the discussion, while the other served as a notetaker. Participants were informed of anticipated risks and provided written informed consent before each FGD began. The primary risk – loss of confidentiality – was minimized by requesting that participants choose a pseudonym for the duration of the FGD and by reminding participants not to share any details about the discussion outside of the FGD. The FGDs were facilitated using a standardized discussion guide developed by the DPP study team. FGDs were conducted in English and/or isiZulu (the most common local language), depending on participant preferences, and audio-recorded. The notetaker also captured key points discussed and documented interactions and nonverbal cues observed during the discussions. FGDs lasted approximately 1.5 to 2 h.

During the FGDs, participants received detailed information about COCs, PrEP, and the potential characteristics of the DPP. The interview guide then explored opinions on (1) current pregnancy risk and avoidance strategies, (2) current HIV risk perceptions and avoidance strategies, (3) PrEP knowledge, attitudes, and perceptions, (4) COC practices, (5) opinions about the DPP, and (6) what needs to be in place for women to be able to use the DPP. A participatory activity also formed part of the FGD, using a minibus as an analogy to elicit participant perspectives on potential facilitators and roadblocks for the DPP. FGDs were transcribed and translated directly into English, as applicable. Debriefing reports were prepared to provide a rapid summary of each FGD.

### Data analysis

An ethnographic content analysis approach was used for the data analysis, [32, 33] which allows categories and themes to flow from the data rather than solely from the researcher’s preconceptions. Data included FGD transcripts, debriefing reports prepared by the data collection team, and analytical memos drafted by data analysts during data collection. FGD transcripts were reviewed, and a code book was developed based on the objectives of the study as well as emergent themes from the data. Transcripts were coded by seven coders on the research team using NVivo software version 1.4 (Lumivero, Burlington Massachusetts, United States, Country). For quality control, 10% of the data were coded by more than one analyst. Weekly team meetings were used to discuss and resolve disagreements in coding and to discuss findings. Once there were no differences in the understanding and application of codes, convergent validity was considered to have been achieved. Study analysts reviewed and summarized code reports and assembled the data into descriptions of attitudes and considerations about DPP. Subsequently, we compared whether the same findings emerged across discussions with COC and non-COC users and across age groups. At every step of the analytic process, findings were reviewed by all analysts to ensure contextual validity.

### Ethical approval

The study protocol was reviewed and approved by the Population Council Institutional Review Board and the University of the Witwatersrand Johannesburg Human Research Ethics Committee. All participants received ZAR300 (~ $20) reimbursement for their time, inconvenience, and expenses commensurate with local guidelines regarding study participation.

## Results

### Study participants

Between March and July 2021, 12 FGDs were conducted with a total of 74 participants (Table 1). Participants were on average 23 years of age, 43% were married or living as married had an average of 1.2 partners in past three months, over half were currently in school, 21% were currently earning an income, 46% reported currently using COCs, and 58% reported currently using PrEP. Participants who were nonCOC users reported either using condoms for HIV and pregnancy prevention or not using any method at the time of data collection.

**Table 1:** Participant characteristics (*n* = 74)

Characteristics	COC-Users (*n* = 34) (%)	Non-COC-Users (*n* = 40) (%)
Mean age, range	24, 16–36 years	23, 16–40 years
Married or living as married	41	43
Average # partners in the last 3 months	1	1
Currently in school	56	50
Earns income	38	8
Currently using PrEP	55	53

### How might the DPP meet women’s prevention needs?

Participants generally felt that use of the DPP would reduce both new HIV infections and unplanned pregnancy.

#### Convenience

Women appreciated the idea of combining the two pills into one for ease of use.

“Yes, I mean PrEP and contraceptives all in one? It makes so much sense because now you don’t have to be taking PrEP and then on top of that you are taking triphasil or oral contraceptives; it just kills 2 birds with one stone, just like that.” [FGD 10, COC-user, 25–40 age group].

In addition, they noted the benefit of not having to go to two separate clinics for PrEP and oral contraceptive pills.

“It’s a good pill for them to get going, Its one pill, it is not two. You also don’t have to go to the clinic two different times for two different pills.” [FGD03, nonCOC user, 16–17 age group].

#### Benefits for AGYW

Women felt that DPP would be more beneficial to AGYW than older women because AGYW are more likely to engage in unprotected sex and potentially have multiple sexual partners. Older women generally commented on young women being irresponsible and rushing into relationships before having a chance to jointly test for HIV. This sentiment was also reiterated by AGYW participants when asked why they consider the DPP to be more beneficial to them, stating *“Because we’re busier than adults, and we like to engage in a lot of things. (FGD05, COC-user, 18–24 age group).”* Another individual in the group noted, “*I just think it’s safer and it will aid teenagers to have sex properly/safer.”* [FGD01*, nonCOC user, 16–17 age group*].

Regarding the prevention of unplanned pregnancy in addition to HIV, the participants noted that the DPP will be beneficial for young women still in school because falling pregnant may force them to drop out of school due to a lack of support for childcare needs from family and partners. Pregnancy is also seen as hindering women’s future plans, and therefore, additional methods to avoid unplanned pregnancy would be welcome. They considered the prevention of pregnancy indication would be valuable in contributing to PrEP adherence. A few young women also reported it would also help reduce abortions, both legal and backstreet, and/or child abandonment by unprepared young mums.

Participants speaking of delayed future plans reported, *“Now, less ladies like teenagers they will not drop out of school anymore because once you are pregnant, you going to have to leave school, go give birth, look after the child and then go back to school and it affects you, …, at least now with this pill, at least …, I don’t have to leave school because of being pregnant.” (FGD03, nonCOC user, 16–17 age group).*

#### Useful for unprotected/condomless sex

An additional advantage of the DPP would be that it would allow for covert use in relationships where the use of safer sex methods, like condoms, is limited or difficult to negotiate with partners. Women stated that men hold greater influence in relationships, both in terms of childbearing intentions and HIV prevention. Women often struggle to voice a lack of readiness to have children if the partner is insistent upon it. Among adult married women, participants noted a desire to use the DPP covertly from their husbands.

Okay, I think with women our age that are married some of them aren’t even allowed to go for family planning or anything but if you take DPP without your partner knowing you will just keep saying that yes you having another child but knowing full well that you’re taking DPP on the other side, because when you’re married you don’t have much of a say it always tell you this is what will happen and you can’t dispute it, also because majority of the married women’s husbands are against family planning [FGD11, nonCOC user, 25–40 age group].

Reporting on covert use of DPP*, “I think it’s something good, especially for married women because married men don’t want to sleep with a condom, so you can even take it without him knowing it”. [FGD12, nonCOC user, 25–40 age group].*

Women also articulated a desire to please their partners. They noted that they were worried about their partners leaving them, which made it harder for them to negotiate condom use.

‘’It will help us by the fear of our boyfriend leaving us, like with this pill now I know that if he says this [he does not want to use condoms], I will say yes, and they will become happy, and I also become happy. No one needs to sacrifice’’ [FGD03, nonCOC user, 16–17 age group].

Participants noted that the DPP could be beneficial in the event of rape, as it could provide dual protection against HIV and unplanned pregnancy.

*“I agree with her … in case you get raped, and the person doesn’t use a condom at all, and that person has HIV, which might affect you, you might get pregnant, and HIV and you know you are safe because you are using this pill that’s preventing HIV and pregnancy. [FDG03, nonCOC user, 16-17 age group]*.”

Several reasons were given for why women might prefer using the DPP over a condom. Many women felt that condoms were likely to break, making them an unreliable form of birth control or HIV prevention tool.

“A condom can obviously burst, and it increases the risk of getting this [HIV] infection, so I think the pill might help because it prevents you from getting infected with HIV.” (FGD02, COC-user, 16–17 age group).

Respondents also mentioned the fear that men could remove the condom during sex without their knowledge.

“We both agree that we’re going to use protection, and then during sex, he makes you do Dog style [sex position], then takes out the condoms while you can’t feel.” [FGD05, COC-user, 18–24 age group]

In addition to fears that men would remove the condom, women were also concerned that men might tamper with or damage the condom without their knowledge with the intention of impregnating women. Women suggested that the DPP would be useful in helping them navigate situations such as this where the man thinks he is getting the upper hand.

“Even if he can damage the condom, is it that he does not know that I am taking the DPP, so to his mind he damages the condom to make me pregnant, so I won’t be pregnant and he will be surprised, and he will be stressed [laughing].” [FGD04, COC-user, 16–17 age group].

“Even these men they break them themselves. Serious, I mean when you look sideways, they make holes on the condoms.” [FGD12, nonCOC user, 25–40 age group]

I would compare them by saying the DPP is safer because isn’t it condoms burst and sometimes boys would use expired condoms because they do not care, they do not check the dates; so, the DPP is good because the boy would be thinking “this girl is a dummy, I’ll just use this condom that’s expired and she would just fall pregnant and have a baby” [FGD02, COC-user, 16–17 age group].

There was an overall sense among women of all ages who were not COC users that condoms were not 100% effective and that the DPP would be more reliable, helping prevent unplanned pregnancies and HIV infection.

“Condoms do help, but as we know that they are not 100% accurate so the pill (DPP) that you guys are about to introduce, it will help a lot to prevent HIV and pregnancy, and the percentage of young girls getting HIV and pregnant will decrease.” [FGD08, nonCOC user, 18–24 age group].

The DPP was noted as potentially superior in level of protection specifically because its protection is not localized, i.e., it protects only the vaginal area and instead provides body-wide protection from HIV and pregnancy.

“Like condoms may be not a 100% safe and now DPP is going to go through your whole body, you will not get pregnant. With condoms, a guy would take a needle and just burst it and you become pregnant, and now with these pills you will not get pregnant because it is in your system, its flowing” [FGD03, nonCOC user, 16–17 age group].

There was a sense among women who were > 25 years and COC users that even if women had the best intentions and wanted to use a condom, whether influenced by relationship dynamics, alcohol, drugs, or emotions, they might be less likely to use a condom during sex.

Reporting on best intentions of condom use being interrupted by emotions, participants stated, *“after the kissing, when you are dizzy and then you want it inside, meaning you would want to have sex without a condom, and you would say “why use a condom”. And they would also say “let me just penetrate a little bit… Let it rest on the thighs” then suddenly you are having the sex [laughing]” [FGD09, COC-user, 25–40 age group].*

Given all the barriers women face around consistent condom use, whether it is forgetting to use one, negotiating their use, fears that the men will refuse to use or are tampering with condoms or the condom itself bursts, the DPP offers an alternative where women are protected from both pregnancy and HIV.

### Potential facilitators and roadblocks for the DPP

**Fig. 1:**
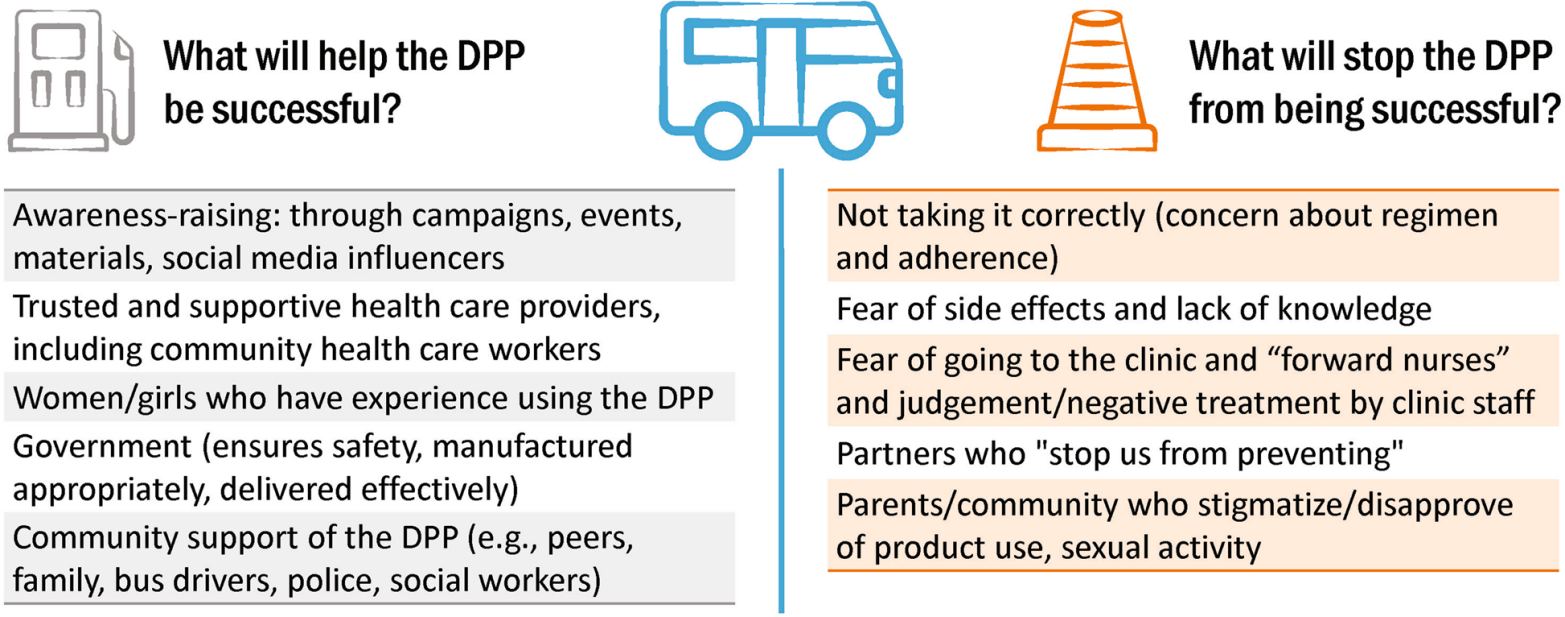
Potential facilitators and roadblocks of the DPP

In the analogy, the minibus represents everything that needs to happen for DPP to be available for women in this community. We asked participants about what would help/facilitate the project moving forward (i.e., all the elements that would enable the DPP to be successful) and what would present a roadblock/prevent the minibus from getting to its destination. Figure 1 shows a summary of this discussion among COC and non-COC users. Within each column, the factors mentioned the most were listed at the top.

As shown on the left side of Fig. 1, potential facilitators of the DPP included broad-based awareness about the DPP, supportive and quality health care, knowing other women who have used the product, knowing the product is safe and has been approved by the government, and community support for use. As listed oOn the right side of the image, potential roadblocks for the DPP included both product-specific (regimen and side effects) and nonproduct-specific factors (negative clinic experiences, partner considerations, and parental/community disapproval).

#### Adherence to daily regimen

Both current COC users and nonusers, as well as women with daily oral PrEP use experience, noted that they might encounter problems with adherence to the DPP’s daily use regimen. They noted that they would struggle with taking it every day and at the same time every day. A group of young women described it as “boring” *(local slang referring to something that one finds, labor intensive or tedious)* to have to take a pill every day. Women were particularly discouraged from learning that if they did not take the DPP exactly as prescribed – same time every day – then the pill would not work.

“Taking a pill every day will be too much work you will forget to take it. And if you take it at the same time, you usually take it OR it will not work.” (FGD08, nonCOC user, 18–24 age group)

Another group of women in their early 20 s who are currently using COC and have used oral PrEP noted that while they are motivated to take the pills, their busy lifestyles at times prevent them from adhering to the required regimen.

“Eish so sometimes you forget, and it’s not that you forget because you want to, sometimes you’re late, I mean you know there’s a pill you have to drink but, I think its forgettable, not the actual pill but, like us as the youth, we have a lot of things that we’re doing, you understand, we have a lot of friends we do quite a lot of things all at once, and we forget about all the important stuff that we need to do. [FGD05, COC-user, 18–24 age group].

Some women expressed interest in a different formulation, particularly a long-acting injectable, to avoid frequent clinic visits and adherence issues related to a daily pill.

*“Okay, I wanted to say something about the DPP; like, because it is pills and we are supposed to take them at the same time, I say it would be better if it’s an injection because you just get injected once after two months; so, the challenge is that one (FGD01, nonCOC user, 16-17 age group).”*

#### Side effects

Nearly all participants were concerned about side effects, particularly those that would have an impact on daily life. Only three women who were current COC users felt the side effects would either not be an issue, or the benefits of the DPP would outweigh the potential side effects. Depression, changes in menstrual bleeding, upset stomach, trouble sleeping, headaches, and dark spots on the skin were seen as worrisome side effects among women.

Uhm as far as I am concerned, depression is a disease, and it needs to be treated so if it falls under the possible side effects and I have depression I might end up hurting myself, doing something bad to myself, even worse is suicidal thoughts, so I’m scared of depression. [FGD04, COC-user, 16–17 age group].

Visible side effects, including weight loss, vomiting, or lethargy, were associated with potential stigma around suspected HIV infection or pregnancy.

Weight loss for me is serious because people will see that you have lost weight and then they start talking and making remarks about me and start assuming that I am HIV positive. [FGD08, nonCOC user, 18–24 age group].

Lack of energy and tiredness. Some parents would be having talks if you mention that you are tired. They would be saying “yeah! You went to sleep around with boys, they have drained all your energy”. Or they would say you are pregnant. [FGD01, nonCOC user, 16–17 age group].

There were a few comments*,* more frequently among non-COC users, regarding past use of and dissatisfaction with other contraceptive methods due to side effects (such as changes to their menstrual cycle and weight loss), that impacted their daily lives and eventually led some women to method discontinuation.

Serious but rare side effects, including liver and kidney damage associated with the PrEP component of the DPP and risk of blood clots associated with the hormones, were seen as concerning. Participants stated that mild kidney problems may lead to one eventually developing severe kidney problems that would require a kidney transplant, with high associated costs, or even lead to death.

“Liver problems, like anything that affects your body also affects you as a person, so just by drinking a pill to prevent certain things, you inadvertently get sick with other things, so no it doesn’t work.” [FGD05, COC-user, 18–24 age group].

Participants had questions about how long DPP side effects would last, with greater length being of higher concern. A few women mentioned a fear that the DPP would be associated with delayed return to fertility and/or future infertility. This was mentioned more frequently among the non-COC users, with comments implying a preexisting belief that contraceptive use, in general, is associated with future infertility.

“Can I ask a question? When you are drinking this DPP a lot, is it going to cause you to be infertile? Or it won’t, when you want to have kids, you will be able to or its going to block you forever? (…) Sometimes those oral contraceptives make some women infertile. So, I want to know the consequences of DPP.” [FGD04, COC-user, 16–17 age group].

Finally, there were a few requests from participants to reformulate the DPP to have fewer side effects.

“They need to reduce the side effects, even if it means adding more chemicals.” [FGD05, COC-user, 18–24 age group].

#### Negative experiences at health facilities

A significant barrier to DPP use that was brought up by women of all age groups was feeling judged by healthcare providers. This was especially common among AGYW, who feared being stigmatized by healthcare workers. Young women reported feeling judged by providers who viewed them as promiscuous or believed they were engaged in sex work because they were seeking prevention services. Several participants also raised concerns about a lack of confidentiality.

When you go to the clinics for prevention, the nurses tell you that you are young, ask you what you are doing when you are this young, ask you who you are sleeping with when you are this young. It’s none of their business, seriously. They are so annoying, which is why we are afraid to go to the clinics for prevention, and we end up falling pregnant. [FGD01, nonCOC user, 16–17 age group].

Some nurses would ask you why you are having sex when you are so young, why are you letting yourself be fooled by boys, and then why do you have to take contraceptives to avoid being pregnant. Others even gossip about us; you would hear them saying this kid likes doing old people’s activities. [FGD04, COC-user, 16–17 age group].

A few women referred to the contraceptive supply shortages that frequently occur in public clinics in South Africa and that have prevented them from accessing their desired method in the past. Women raised that these negative experiences at public clinics, namely, bad attitudes from nurses and supply shortages, will likely influence access to and uptake of the DPP. Several participants described overarching service delivery challenges that would be barriers to DPP delivery, as articulated by this participant:

“I am concerned. I mean theoretically, this is wonderful, it is stepping the right direction, but as long as we still have shortage of supply in our clinics with these pills because as long as there is no supply, then we are not preventing anything. And as long as we have staff members who behave this way in our clinics, then those pills will stay in those cupboards, and they won’t prevent anything. So, we need to change the attitudes in the health fraternity, your colleagues, and then make sure that there is supply”. [FGD09, COC-user, 25–40 age group].

#### Partners and permission to use contraceptives.* Relationship power dynamics*

Preferences for prevention product use were a key consideration mentioned by women and girls. Women noted that they should be able to make autonomous decisions but understood how their partners’ behaviors influenced their vulnerability to HIV and unplanned pregnancy.

“…they [women] do not need anyone’s permission because it only concerns you; if your man doesn’t sleep at home, it affects you if you were to get HIV, so it’s your responsibility to protect yourself.” [FGD01, nonCOC user, 16–17 age group].

At the same time, respondents noted that women, especially those in marital relationships (defined broadly to include formal or informal marriage and cohabitation), in most cases need support and permission from their partners to be able to use contraceptives. Women stated that partners may be resistant to DPP because it conflicts with their desire and expectation for children. A group of married women in their mid-30 s who are not currently using oral contraceptives but highlighted that women their age use condoms and injectable contraception, noted:

“I think with women our age that are married some of them aren’t even allowed to go for family planning or anything but if you take DPP without your partner knowing you will just keep saying that yes you having another child but knowing full well that you’re taking DPP on the other side, because when you’re married you don’t have much of a say…, also because majority of the married women’s husbands are against family planning…I think when a man marries a woman, he feels that he owns her, and he feels you are there to make kids for him and to extend the family’’ [FGD11, nonCOC user, 25–40 age group].

Additionally, a group of adolescent girls also mentioned their partners being concerned about their use of contraceptives, with them being worried that contraceptives may affect their partners’ prospects of pregnancy in the future. Additionally, reports from some participants expressed male partner permission, forming part of contraceptive use decision making. Participants who are current COC users noted:

“My boyfriend also allows me to use contraceptives, right? However, he sometimes mentions that it could happen that we are unable to have children in the future because I am taking contraceptives.” [FGD02 COC-user, 16–17 age group].

However, many women noted that they resorted to covert use of contraceptives to avoid unplanned pregnancies, as they were often being pressured by partners to have children.

“Yes, he doesn’t know. He knew before we stated dating until he told me that he wanted a baby I said okay, but I am still taking the pills I have to hide them, put them in my bra or drink them at night, that is my challenge with my partner even my previous partner wanted a baby, but he was not forcing me like my current partner. Okay [before] I pushed myself for the injection even though it makes you feel pregnant, you eat a lot. Now I am taking the pill and I am hiding them from my partner.” [FGD06 COC-user, 18–24 age group].

This demonstrated the autonomy that some empowered women exercised in their own sexual reproductive health decision making. Given this pressure and expectation of childbearing, women noted that they would find disclosure of the DPP a challenge with their marital or main partners, and some may opt for more discrete use.

#### Family and community stigma around adolescent sexuality and HIV

Adolescent participants mentioned that parents and family members can be judgmental in relation to contraceptive use. Reporting on her family’s reaction to her contraception use, one participant stated,

“With my family, they cannot understand the fact that I am on contraceptive pills; only my aunt understands.” [FGD02 COC-user, 16–17 age group].

Within the community at large, women stated there is stigma around AGYW being sexually active, in general, which impacts young women’s ability to access sexual and reproductive healthcare, including contraception and HIV prevention services. AGYW seeking HIV prevention services are further stigmatized because there is an assumption that they are already HIV-infected and taking antiretrovirals (ARVs) for prevention.

“The thing is we are living in a toxic society, very toxic. So, immediately when you are drinking such pills, they will start to think that you are positive [referring to being HIV positive] or saying she started sleeping around with boys, community is not it” [referring to community opinions being negative]. [FGD04 COC-user, 16–17 age group].

With the stigma, we have got stigma in communities. Uhm, you are pregnant, the oldies from 60 years old going up would look at you like this “waywo” [child] who is forward and having sex. That’s number one, they lose all respect for you because you are pregnant, while they forget that they also had children at the same age as you. [FGD10 COC-user, 25–40 age group].

In relation to how this will impact the DPP, a majority of the women noted that once people in the community learn that the DPP is for both HIV and pregnancy prevention, they will assume users already have HIV and are taking ARVs.

“Exactly, they will think that now that you’re taking this pill you’re up to no good, you’re not playing it safe. Even if they don’t know whether you’re in a relationship or not, as long as they know what this pill does, they will assume the worst…They will think its ARVS, and I think other people’s opinions shouldn’t matter when it comes to your health, because you know the truth that its DPP and not what they might think it is”. [FGD07 nonCOC user, 18–24 age group].

#### Where to determine the DPP and where to obtain it

Participants were asked to share their opinions about where the DPP should be made available. Many participants emphasized the importance of awareness-raising campaigns and listed an array of locations and ways of determining the DPP. Table 2 lists participants’ suggestions for where people should be able to obtain information about the DPP and where it should be provided.

**Table 2:** Suggested locations for information and distribution of the DPP

Location	**Information**	**Distribution**
Clinics	**X**	**X**
Chemists	**X**	**X**
Schools	**X**	**X**
Internet	**X**	
Pamphlets	**X**	
Entertainment venues (clubs, taverns)	**X**	**X**
Friends/people using	**X**	
Traditional healers	**X**	**X**
News	**X**	
Radio	**X**	
TV adverts (older participants)	**X**	
Facebook/Social media	**X**	
Cinema (younger participants)	**X**	
Door-to-door campaigning (older group)	**X**	
Churches (older)	**X**	
Taxi ranks (older)	**X**	
Vending machines		**X**
Bathrooms		**X**
Brothels		**X**
Mobile clinics		**X**

When asked what they thought about the name, “dual prevention pill,” some participants offered alternatives. In the South African context, the word “prevention” typically refers to contraceptives and may imply early sexual debut and contribute to stigma; therefore, they suggested replacing the word “prevention” with “protection.”

“If you can just remove the “prevention” part. Once people hear the word “prevention”, they will start talking. Dual protection pill [FGD08 nonCOC user, 18–24 age group]

Additionally, participants felt that having trusted and supportive healthcare providers would facilitate uptake and use of the DPP. Women of all ages said the DPP should be provided at public clinics, despite some of their experiences for some rude and judgmental nurses, as these clinics are accessible, free, and generally where women go to get health services for free.

“From the clinic’s ma’am, we would like to get it [DPP] from the clinics… Because clinics are where we get our help from”. [FGD01, nonCOC user, 16–17 age group].

“I think clinic, hospitals, pharmacies, anywhere that you can get birth control now, that’s where it should be available and for free because I’m sure PrEP is not for free, there’s only certain places where you can get PrEP its only at LGBTQ clinics that you can get PrEP or else you would need to pay for it.” [FGD11, nonCOC user, 25–40 age group].

There were many proposals to offer the DPP in chemists or pharmacies where participants felt they would not be judged and would not have to wait in long queues. However, they also acknowledged that having to pay for the DPP would create a financial barrier for those who cannot afford the product outside the public sector.

“I also wanted to say that because at clicks they also give ARVs. There’s a friend of mine that goes there to collect, so why can’t we also go to the pharmacies instead of going to the clinics because they even give us nasty attitude as if they get paid for them to give us those pills. Therefore, we have to go to the pharmacies. [FGD10 COC-user, 25–40 age group].

“I also think that the pharmacy is good because isn’t it the staff in clinics is judgmental? So, they would be asking you what you want to do with this pill; they won’t tell you how the pill is taken, they would just tell you that you can take it with anything. So, the pharmacy is good, but they must sell it at a lower price.” [FGD01, nonCOC user, 16–17 age group].

There were mixed views on the acceptability of the distribution of the DPP at schools. Some felt that schools would overcome stigma-related barriers faced by young people at clinics, whereas others felt that schools were not appropriate because they did not have the medical staffing needed.

“You know why I am saying in school, because I know there are lot of learners who are afraid of going to the clinic or pharmacies because of money, some schools can even provide them with the pills because most girls already have boyfriends at schools it will be much easier even than going to the clinic while they can get it there or learn about it, especially in high school it will be much easier because they can’t stay out of school then go to the pharmacy or to the clinic”. [FGD08 nonCOC user, 18–24 age group].

“Sorry, I think I disagree on the high school part. The reason why we get pills from the clinics is because it is a medical institution and a school is not a medical institution; the reason why you have to go check your high blood pressure, check your sugar before they give you your medication is for a reason. So they can’t just hand out pills and say “here, take this DPP””. [FGD10 COC-user, 25–40 age group].

Participants suggested promoting the DPP through the media, TV, and newspapers, especially targeting older generations who may be judgmental.

“I think from older people as well because older people are so judgmental ma’am. Therefore, if it gets published in places like the T.V.s, the internet and newspaper; they would read them of course and I think also because a lot of older people watch T.V a lot. They also love the internet and watch the news, so they would be updated and know what the DPP does, and then they would be able to advise us.” [FGD01, nonCOC user, 16–17 age group].

Younger participants felt they would want to learn about the DPP before going to the clinic so that they could go already knowing to ask for it. Having information available on the internet would also facilitate discussion with parents.

“(…) with DPP, it would be best to show it on the internet because now if you say mom, I'm going to be using DPP, you should have enough information about it to say mom check on the internet and you will see what I'm talking about”. [FGD03, nonCOC user, 16–17 age group].

#### Hearing other women who have experience with the DPP

Many participants felt that word of mouth would be best, noting that they would prefer to hear about other women’s experience using the DPP before they try it themselves. To address side effect concerns, for instance, women mentioned that if they hear about other women’s experience with use of the DPP and side effect management, they will be more comfortable with potential DPP side effects.

“The project needs people who know more about the DPP so that they can run and distribute the product; there should also be people with experience, people who have used the DPP before and have never had side effects and those who have had side effects so that if you experience them so that they can tell you, if you experience them, how they experienced them and this is what I did. This is so that people would be comfortable enough to say that okay I can use it, and the side effects won’t be as bad.” [FGD01, nonCOC user, 16–17 age group].

#### Government approvals

Participants (both COC users and non-COC users) pointed to governmental systems as crucial sources of legitimacy for potential DPP consumers. The government has long been viewed as a driving force behind any new development in healthcare services, be it as a funder, a manufacturer or distributor of the service. Similarly, the government was viewed as a key player in the success of DPP implementation with DPP awareness campaigns run by the government, as suggested throughout the discussions with the participants. Additionally, clinics run by the government are seen as the main access point to free healthcare services, and this is true as access to FP as well as HIV prevention and treatment services, such as PrEP and ART, have been championed and provided primarily through government local clinics.

#### Community support

Hand-in-hand with awareness raising campaigns, participants felt that there needed to be broad-based community support for DPP use. Participants mentioned peers, bus drivers, and social workers. One participant mentioned that it would be important to inform the police about the DPP so that they can tell the police that the DPP is not an illicit drug.

“I think that others should be the doctor and the policemen like she said, just in case, you know when there are roadblocks, you get pulled over and they start searching you so for them to be sure it's not drugs, the nurse and doctor will have proof that it's something for the clinics and not drugs. And the police would be there to speak to the Metro and tell them no we are escorting them, so the tablets will go faster”. [FGD03, nonCOC user, 16–17 age group].

## Discussion

We set out to understand South African women’s interest in and opinions about the DPP, and to gather their suggestions for its potential introduction. Overall, we found that women want an MPT, such as the DPP, that combines HIV and pregnancy prevention. Interest in the DPP was based primarily on it being deemed easy to use, more reliable than condoms, and facilitating streamlined services for HIV and pregnancy prevention. On the other hand, women indicated that they anticipate challenges taking a daily pill. Additionally, women highlighted persistent challenges with women’s current access to and support for use of PrEP and contraceptives, which should be considered for MPT development and introduction. Our data point to women’s need for more MPT options. It also brings to light the need for more research into the development of MPTs and careful consideration of social and health system factors impacting introduction.

A key product-related concern noted by the participants was regarding adherence. Women indicated that daily pill taking may be a challenge, echoing similar sentiments from recent PrEP studies [34]. Data from the DPP study indicate that it will be important to consider different MPT formulations, considering that many participants suggested a long-acting injectable for pregnancy and HIV prevention, highlighting a need for choices in HIV and pregnancy prevention options.

Concerns about DPP side effects emanated from participants’ experiences with PrEP or COCs, along with learning about potential side effects during participation in the DPP formative study. Such concerns have the potential to limit uptake of DPP. A prospective study conducted in South Africa (CAPRISA 082) that offered PrEP as part of a comprehensive HIV prevention care package found that side effects contributed the most to early PrEP discontinuation, accounting for up to fifty percent of the study participants who had been initiated on PrEP [35]. In an ancillary study to the ECHO trial conducted in South Africa, similar discontinuation patterns were observed; although fewer than fifty percent discontinued PrEP use in this study, the primary reason for discontinuation was side effects [36]. Data from the DPP formative research study align with previous PrEP studies, in which participants are concerned about side effects that affect their daily lives, as well as more serious side effects that are rarer or may happen in the future. Ideally, MPTs could be developed to have fewer side effects; however, regardless of the side effect profile, information about anticipated side effects and supportive counseling to help women cope with side effects are important parts of the service delivery package for the DPP and future MPTs. Study participants also highlighted the importance of hearing directly from other women about their product use experiences to learn about how they had managed any potential side effects. Subsequent DPP roll-out should carefully consider support strategies for DPP use (e.g., support groups) as the product is first introduced.

Perceived healthcare provider attitudes are a key potential barrier noted in our study, specifically in relation to the stigmatization of young women when seeking services at their local clinics and from participant responses, which has an overarching impact affecting services beyond sexual and reproductive health. Considering that local clinics provide free and accessible health services and are the largest point of access for future MPTs, this barrier forms a major hurdle in access to services. DPP introduction alone will not change the experiences of women facing stigma when accessing services; however, we need to address the attitudes of healthcare providers, particularly toward younger clients, for successful implementation of the DPP. Taking a whole system approach has been shown to be useful in addressing HIV-related stigma, whereby facilities create awareness among providers, nurses, counselors, registration desk staff, security guards and any other cadre who interacts with clients of the impact they have on individuals as they seek healthcare services [37, 38]. Similarly, Lanham and colleagues [39] used results from a mixed methods study encompassing a survey followed by qualitative interviews with PrEP healthcare providers to assess their primary concerns in providing PrEP to AGYW, with an end goal of addressing these concerns through training. This work provides another potential means to address healthcare providers by providing training targeted at healthcare provider needs.

Partner approval of HIV and pregnancy prevention methods is another potential barrier to DPP use, particularly among women who are married or cohabiting. Some male partners are resistant to contraceptive use, as they expect to control or at a minimum to be involved in decisions around the number and timing of children. In addition, the use of HIV prevention products has been cited to raise questions of trust in the relationship. Such cases of male partner resistance to the use of both HIV and pregnancy prevention methods have also been noted in other research. For an example, a qualitative study conducted in two provinces with high HIV infection rates in South Africa, Mpumalanga and Kwa-Zulu Natal, highlighted the use of PrEP as being associated with suspicions infidelity and lack of trust by the male partners [40]. Education initiatives directed toward male partners about the DPP and broader male engagement strategies [41], may be needed to facilitate women’s ability to use the DPP, as echoed by all participants in our study.

Community opinions on the use of HIV and pregnancy prevention options, rooted in social, cultural and religious beliefs (e.g., product use insinuating early sexual debut, deemed culturally and religiously inappropriate), was another consideration for the introduction of MPTs such as DPP. In addition, community misconceptions on pregnancy prevention products also exist (e.g., that use impacts fertility later in life). Misconceptions of HIV prevention products being seen as HIV treatment contribute to stigma in use and form a barrier to uptake. Barriers identified in our study are also present in other PrEP studies. For instance, insights from Masibambisane, a qualitative study aimed at likely PrEP users, conducted in Durban, South Africa, highlighted among other potential barriers to PrEP use, the assumption of promiscuity by fellow community members if one uses PrEP [16]. Another contributor to stigma which in turn becomes a barrier to uptake, that would need to be considered in the introduction of products like the DPP, stems from how PrEP was introduced in a country context. In South Africa, for instance, PrEP was introduced through government backed programs as a priority HIV prevention method for those deemed at risk of HIV infection, including AGYW and sex workers. This classification came with any potential PrEP users being perceived as participating in risky sexual behavior. This context of PrEP introduction also potentially contributed to stigma experienced by younger women, seeking PrEP services from their local clinics. A qualitative component, part of a larger clinical trial HPTN 082, assessing the characteristics of women who accepted or declined PrEP at study enrollment, conducted in Johannesburg, South Africa, highlighted the impact of stigma emanating from beliefs around risky sexual behavior in PrEP users, impacting both disclosure of product use in PrEP users and adherence to use [42]. From the discussions held with the women in our study, it is evident that the DPP on its own will not resolve the currently existing community challenges. Concerted community engagement efforts will be needed to generate demand, dispel rumors and raise awareness on the DPP. These should comprise of both multimedia approaches alongside in-person health service center-based sessions and workshops targeting healthcare providers, community members and potential DPP users. Additionally, these should be supported by both government and non-governmental organizations who are the current main providers of sexual health services. Moreover, these approaches have been highlighted in past research as having the potential to create awareness on HIV prevention services [43, 44]. Important to note is that these services, in the process of creating awareness, should also work at reframing PrEP from being defined as being for those at risk to being a product for HIV prevention that can be used by all who see a need for it, regardless of sexual behavior [42, 43, 45, 46].

### Limitations

Our research has several limitations. Firstly, this was a small cross-sectional research study about a hypothetical product. Although we provided detailed information to participants about the likely characteristics of the DPP, participants were unable to see the actual product or know its exact attributes. Subsequent research should examine women’s experiences with the actual DPP when available. Additionally, the FGD participants may have shared experiences in accessing healthcare services, resulting in convergence of opinions about local health services as participants from within the same area of residence were recruited for the study. We only compared the perspectives of COC and non-COC users; it may be important to investigate perspectives about the DPP among women who have an unmet need for contraception or are not satisfied with their current contraceptive method to understand how the DPP may or may not fit their protection needs.

## Conclusion

In this study, women seemed to report that the DPP is a potentially viable MPT option for women in Johannesburg that could improve PrEP uptake and provide another option for pregnancy protection. Implementation programs related to the DPP need to consider both product-related and nonproduct-related potential barriers to DPP uptake and use. DPP uptake will be supported by clear, accurate, and specific guidance on the product, broad-based awareness raising and community support, trusted and supportive care provision, and connections with women who have experience with the product. Additionally, interpersonal (partner perspectives) and social (parents/community perspectives) implications of use will need to be considered to ensure women are able to use this innovative product, once available.

## Data Availability

The datasets used and analysed during for the DPP study, are available from the corresponding author on reasonable request.
